# Aortic Regurgitation in a Patient with Rheumatoid Arthritis/Systemic Lupus Erythematosus Overlap Syndrome (Rhupus): Case Report and Review of Literature

**DOI:** 10.3390/jcdd12100408

**Published:** 2025-10-16

**Authors:** Mislav Radić, Hana Đogaš, Tina Bečić, Petra Šimac, Ivana Jukić, Josipa Radić, Damir Fabijanić

**Affiliations:** 1 Department of Internal Medicine, Division of Rheumatology, Allergology and Clinical Immunology, Center of Excellence for Systemic Sclerosis in Croatia, University Hospital of Split, 21000 Split, Croatia; psimac@kbsplit.hr; 2 Internal Medicine Department, School of Medicine, University of Split, 21000 Split, Croatia; jradic@kbsplit.hr (J.R.); dfabijan@mefst.hr (D.F.); 3 Department of Neurology, University Hospital of Split, 21000 Split, Croatia; hdogas@kbsplit.hr; 4 Cardiovascular Disease Department, University Hospital of Split, 21000 Split, Croatia; tbecic@kbsplit.hr; 5 Internal Medicine Department, Gastroenterology Division, University Hospital of Split, 21000 Split, Croatia; ivana.jukic@ozs.unist.hr; 6 Faculty of Health Sciences, University of Split, 21000 Split, Croatia; 7 Department of Internal Medicine, Division of Nephrology, Dialysis and Arterial Hypertension, University Hospital of Split, 21000 Split, Croatia

**Keywords:** rheumatoid arthritis, systemic lupus erythematosus, overlap syndrome, valvular heart disease, aortic regurgitation

## Abstract

Background/Objectives: Cardiovascular diseases (CVDs), including valvular heart disease (VHD), remain the leading cause of morbidity and mortality in patients with rheumatoid arthritis (RA) and systemic lupus erythematosus (SLE). Case Presentation: We report a rare case of a woman in her fifth decade of life diagnosed with overlap syndrome (RA and SLE), in whom transthoracic echocardiography (TTE) and transesophageal echocardiography (TEE) revealed a degenerative altered bicuspid aortic valve with moderate aortic regurgitation (AR) and preserved left ventricular systolic function. The patient presented with a recent history of continuous, moderate chest discomfort and progressive exertional dyspnea, along with a mild elevation in highly cardioselective enzymes. AR was confirmed as the cause of her symptoms, rather than acute coronary syndrome or heart failure. Conclusions: This case highlights the potential contribution of chronic systemic inflammation in RA/SLE to the pathogenesis of AR, an extra-articular manifestation associated with adverse clinical outcomes. These findings support the routine use of echocadiography in rheumatologic patients as a key strategy for early detection and prevention of life-threatening CV complications.

## 1. Introduction

Overlap syndromes are inflammatory rheumatic conditions in which a patient meets the classification criteria for two or more autoimmune rheumatic diseases (ARDs). Due to the lack of universally accepted classification or diagnostic criteria, identifying these rare conditions remains a considerable clinical challenge. The most frequently observed combinations of overlap syndromes include:

- SLE (Systemic lupus erythematosus) + RA (Rheumatoid arthritis): Characterized by features of both diseases, including erosive arthritis and lupus-specific autoantibodies.
- Systemic Sclerosis (SSc) + PM/DM (Polymyositis/Dermatomyositis): Often presents with skin thickening and proximal muscle weakness, along with overlapping serologies.
- SLE + SSc: Exhibits clinical and immunological features of both vasculopathic and fibrotic processes.
- RA + Sjögren’s Syndrome (SjS): Common in RA patients, especially those presenting with sicca symptoms; frequently associated with anti-Ro/SSA and anti-La/SSB antibodies.
- SLE + SjS: Another frequent combination, sharing immunological markers and glandular involvement.
- RA + SSc: May lead to enhanced risk of cardiovascular (CV) and pulmonary complications due to the additive inflammatory and fibrotic burden.
- RA + PM/DM: Presents with arthritis and myositis; myocardial involvement is more likely in such patients.
- Mixed Connective Tissue Disease (MCTD): Features overlapping characteristics of SLE, SSc, and PM/DM; defined by high titers of anti-U1-RNP antibodies.

Overall, overlap syndromes are less prevalent than individual autoimmune diseases. Among them, MCTD is often considered a distinct clinical entity and is the most commonly reported, with a prevalence estimated at approximately one-twentieth that of SLE. Overlapping features are especially common in patients with SLE and RA. However, current classification criteria often perform poorly in such scenarios, particularly when disease features evolve gradually over time [1,2,3,4,5]. “Rhupus,” or RA/SLE overlap syndrome, remains a rare, underrecognized, and poorly defined condition characterized by a combination of clinical and serological markers from both diseases [6]. To date, no validated classification criteria, standardized treatment protocols, or longitudinal studies exist for rhupus, leaving its true epidemiological burden unclear [7]. The prevalence of rhupus is estimated to range from 0.01% to 2% among patients with SLE. In approximately 80% of cases, RA precedes the onset of SLE, although both conditions can occasionally present simultaneously. A hallmark clinical feature is symmetrical polyarthritis, particularly affecting the small joints of the hands, closely resembling RA. Notably, patients with rhupus tend to exhibit fewer major organ manifestations compared to those with classic SLE. Specifically, they have a lower incidence of malar rash, neuropsychiatric lupus, and lupus nephritis. In contrast, serological profiles often reveal elevated titers of rheumatoid factor (RF) and anti-cyclic citrullinated peptide antibodies (ACPA), while the prevalence of anti-double-stranded deoxyribonucleic acid antibodies (anti-dsDNA) and anti-Smith antibodies appears similar to that seen in SLE. Treatment typically involves low- to moderate-dose corticosteroids in combination with conventional synthetic disease-modifying antirheumatic drugs (csDMARDs). For patients who exhibit inadequate response, escalation to immunosuppressive agents or biologic therapies may be warranted [8,9,10,11].

**Figure 1:**
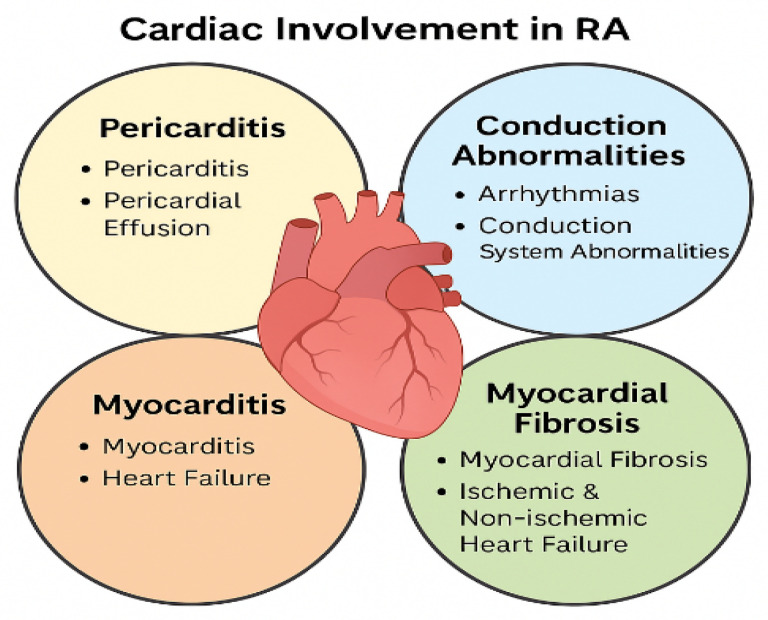
Key forms of cardiac involvement in rheumatoid arthritis (RA), including pericarditis, myocarditis, conduction abnormalities, and myocardial fibrosis. These manifestations may contribute to both ischemic and non-ischemic heart failure and often remain subclinical, underscoring the need for proactive cardiovascular assessment in RA patients.

When RA coexists with conditions such as SLE, SSc, PM/DM, or SjS, the risk of CV complications is significantly heightened. Cardiac involvement in these patients is both diverse and often insidious, encompassing pericarditis, myocarditis, myocardial fibrosis, conduction system abnormalities, arrhythmias, and both ischemic and non-ischemic heart failure (Figure 1). In clinical practice, recognizing patterns of cardiac pathology associated with specific RA overlap syndromes can aid in earlier diagnosis and targeted monitoring. For instance, rhupus, frequently presents with pericardial inflammation, non-bacterial Libman–Sacks endocarditis, and pulmonary hypertension. When RA overlaps with SSc, myocardial fibrosis and conduction defects are common, often contributing to right ventricular dysfunction. In overlaps with PM/DM, myocarditis and dilated cardiomyopathy may occur, sometimes alongside skeletal muscle involvement. In cases where RA coexists with SjS, pericardial effusion, QT interval prolongation, and autonomic dysfunction may be observed. MCTD overlapping with RA may present with a range of findings including pericarditis, pulmonary arterial hypertension, and combined fibrotic cardiac lesions. To support clinicians in evaluating these complex presentations, a visual schematic—depicting anatomical regions of cardiac involvement (e.g., pericardium, myocardium, conduction system, valves) alongside characteristic pathological findings—could serve as an effective diagnostic aid [12,13,14].

Contemporary clinical guidelines underscore the importance of proactive CV screening in this patient population. The 2017 update of the European League Against Rheumatism (EULAR) recommendations on CV risk management in inflammatory joint diseases advises annual CV risk assessment for all RA patients, including blood pressure monitoring, lipid profiling, and evaluation of modifiable risk factors such as smoking. Additionally, they advocate for tight control of inflammatory activity using disease-modifying antirheumatic drugsAPS, along with routine use of electrocardiogram (ECG) and echocardiography (ECHO) in symptomatic patients or those with long-standing disease or elevated inflammatory markers. In patients with suspected myocardial involvement or unexplained cardiac symptoms, advanced imaging such as cardiac magnetic resonance (CMR) or computed tomography (CT) angiography may be warranted [15]. Similarly, the 2021 position paper from the European Society of Cardiology (ESC) supports systematic screening for subclinical cardiac involvement in patients with ARDs, particularly those with overlap features. This includes the application of sensitive imaging techniques, such as speckle-tracking ECHO and CMR, to detect myocardial strain abnormalities or fibrotic remodeling at an early stage. The document further emphasizes the importance of interdisciplinary collaboration between rheumatologists and cardiologists to guide comprehensive and individualized care for these high-risk patients [16]. Incorporating these evidence-based recommendations and proposed visual tools into clinical workflows and educational resources may enhance early detection and optimize management strategies, ultimately improving CV outcomes in patients with RA and its overlap syndromes.

Cardiac involvement in RA can be divided into two main types: rheumatoid granulomatous lesions and non-specific inflammatory changes. Valvular heart disease (VHD) has been observed in approximately 30% of RA patients based on ECHO and autopsy data, with mitral regurgitation appearing more frequently than other forms. Aortic root pathology, including aortitis and aortic regurgitation (AR), has been rarely reported in RA but may represent a severe disease complication [17,18]. Cardiovascular diseases (CVDs) remain a leading cause of morbidity and mortality in patients both with RA and SLE. Reported incidence of CVDs in SLE ranging from 8% to 15%. In contrast to RA, where pericarditis is the most common cardiac manifestation, VHD dominates in SLE [19,20]. Evidence on valvular heart involvement in patients with RA and SLE remains limited. However, undiagnosed VHD may significantly impact patient survival by contributing to the development of heart failure and poor clinical outcomes.

Most drugs used to treat inflammatory rheumatic diseases exert a favorable CV profile by suppressing chronic inflammation, thereby reducing the risk of comorbidities. However, certain therapies may also introduce CV hazards. Glucocorticoids, particularly at prolonged or higher doses, are well known to aggravate traditional risk factors such as hypertension, dyslipidemia, weight gain, and glucose intolerance, ultimately accelerating atherosclerosis. Some targeted therapies have also been linked to increased CV risk, while others require caution in patients with pre-existing heart disease [21]. These considerations are particularly relevant in patients with overlap syndromes, in whom treatment options are often limited by inefficiency to cover all affected domains.

This case report aims to highlight an unusual manifestation of VHD in a patient with rhupus and to explore its diagnostic challenges and therapeutic considerations within the context of clinical practice.

## 2. Case Report

We describe a rare case of a 54-year-old female patient diagnosed with overlap syndrome comprising seronegative RA, classified per the 1987 American Rheumatism Association revised criteria, and SLE, which satisfied both the 1997 American College of Rheumatology revised criteria and the 2019 EULAR/ACR classification criteria, in whom transthoracic echocardiography (TTE) revealed a degeneratively altered bicuspid aortic valve accompanied by moderate AR [21,22,23,24]. Notably, the patient presented with preserved left ventricular ejection fraction, indicating maintained systolic function; however, this does not exclude underlying diastolic dysfunction or myocardial fibrosis, both of which may adversely affect prognosis. The patient has been followed in our rheumatology clinic since 2001 due to rhupus. Over the years, she received multiple csDMARDs, including hydroxychloroquine, methotrexate (MTX), and leflunomide, but all were discontinued due to either adverse effects or insufficient response. Long-term glucocorticoid therapy was continued at low-to-moderate doses. Between 2012 and 2024, the patient received rituximab (RTX), administered biannually to annually at a cumulative dose of 2 g per cycle (divided into two infusions 15 days apart). RTX was withdrawn following the emergence of mucosal ulcerations, recurrent epistaxis, and infections. Moreover, there was a high suspicion of localized inflammation involving the medial epicondyle of the right femur and the right knee prosthesis. These findings were excluded by autologous leukocyte-labeled bone scintigraphy.

The patient has a long-standing history of arterial hypertension and hyperlipidemia. She also has a history of tobacco use, having smoked 10 cigarettes daily for over two decades, yielding a cumulative exposure of more than 10 pack-years. There is no known family history of CVDs. Since 2019, she has been hospitalized on seven occasions due to microcytic, iron-deficiency anemia, chronic diarrhea, exertional dyspnea, and intermittent chest pain. Gastrointestinal and CV evaluations were pursued. Upper and lower endoscopy and abdominal contrast-enhanced CT scans were unremarkable. Multi-slice computed tomography (MSCT) angiography revealed a calcified atherosclerotic plaque with 40–50% luminal stenosis of the proximal right subclavian artery. Coronary angiography ruled out obstructive coronary artery disease and acute ischemic events, revealing only tortuous coronary arteries, a finding generally regarded as a congenital variant rather than a result of arterial hypertension. TTE revealed no significant valvular dysfunction. Twenty-four-hour Holter monitoring showed no relevant bradyarrhythmias or atrioventricular conduction abnormalities.

**Figure 2:**
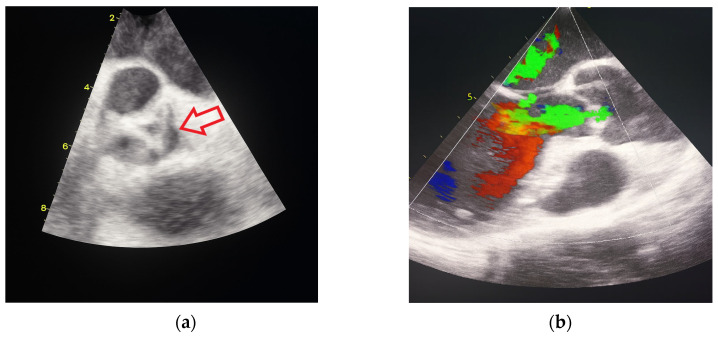
Transesophageal heart ultrasound 2D imaging: (**a**) degeneratively changed bicuspid aortic valve with rudimentary left coronary cusp (red arrow). (**b**) 2D visualization of moderate aortic regurgitation in the context of a pathologically altered aortic valve.

**Figure 3:**
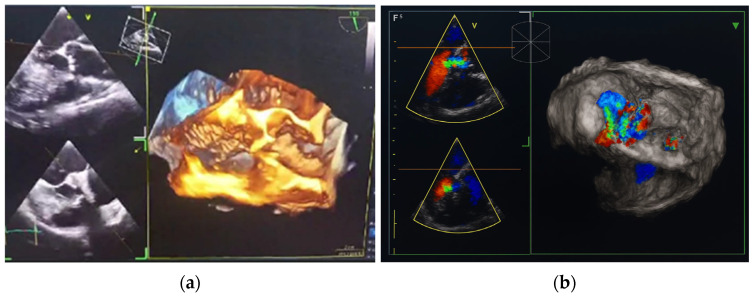
Transesophageal heart ultrasound 3D imaging. (**a**) 3D reconstruction of the aortic root and ascending aorta. (**b**) 3D Color Doppler imaging of aortic regurgitation.

In October 2024, the patient was admitted to our Division of Rheumatology and Immunology due to exacerbation of her underlying overlap syndrome, demonstrating moderate to high disease activity: Disease Activity Score 28-ESR was 4.95 and Systemic Lupus Erythematosus Disease Activity Index was 6. Clinical symptoms included continuous moderate chest discomfort and worsening dyspnea upon exertion. She denied gastrointestinal or respiratory complaints, fever, or systemic signs of infection. Her long-term pharmacotherapy regimen included prednisone 7.5 mg/day, pantoprazole 40 mg/day, atorvastatin 20 mg/day, cholecalciferol 20,000 IU weekly, furosemide 40 mg/day, and lercanidipine 10 mg/day. Vital signs on admission were stable: blood pressure 140/90 mmHg, heart rate 76 bpm, and temperature 36.7 °C. Cardiovascular auscultation revealed a grade 2/6 systolic murmur over the precordium. Musculoskeletal examination was notable for bilateral wrist synovitis, bilateral shoulder pain with restricted range of motion, right knee arthralgia with limited mobility, and fixed flexion deformities of the right-hand fingers and left elbow. There was no clinical evidence of peripheral edema. Laboratory evaluation revealed: leukocytes 6500/µL, hemoglobin 88 g/L, platelets 188,000/µL, C-reactive protein (CRP) 11.7 mg/dL, erythrocyte sedimentation rate (ESR) 50 mm/h, hypocomplementemia (low C3 and C4), serum urea 4.9 mmol/L, and serum creatinine 144 µmol/L. Immunological testing showed negative RF and ACPA. Antinuclear antibodies (ANA) were positive at a titer of 1:160 with a homogeneous nuclear staining (AC-1 pattern). Anti-dsDNA was markedly elevated (86 IU/mL), and antiphospholipid antibodies (aPL), including lupus anticoagulant (LA), were triple positive. All other extractable nuclear antigens tested negative. High-sensitivity troponin I was elevated (84.0 ng/L), whereas N-terminal pro b-type natriuretic peptide (NT-proBNP) remained within normal limits (68.5 ng/L), and ECG findings were unremarkable. Due to persistent anemia, dyspnea, and dysphagia, repeat esophagogastroduodenoscopy was performed, confirming histologically proven erosive Helicobater pylori-negative gastritis. Repeat MSCT coronary angiography again ruled out acute coronary syndromes. Repeat TTE revealed a degenerative bicuspid aortic valve with a rudimentary left coronary cusp and moderate AR (aortic valve area: 2.4 cm^2^; vena contracta: 4 mm). Transesophageal echocardiography (TEE) confirmed these findings and offered a more detailed evaluation of valvular morphology and the severity of AR (Figure 2 and Figure 3).

## 3. Discussion and Review of Literature

Notably, this case represents the first documented occurrence of AR in a patient with RA/SLE overlap syndrome in the setting of a bicuspid aortic valve, a congenital cardiac anomaly. It is also possible that long-term treatment for the overlap condition contributed to accelerated degenerative changes of the aortic valve. Importantly, left ventricular systolic function was preserved. To the best of our knowledge, only a limited number of similar cases have been described in the literature. Valvular heart involvement in RA is a recognized but often underappreciated manifestation, frequently driven by chronic inflammation. This inflammatory process may result in fibrosis and, in some cases, calcification of the valvular apparatus. The most characteristic valvular heart lesion in RA is rheumatoid granulomata. Histologically, these lesions resemble subcutaneous nodules and may develop within valve cusps, contributing to valvular heart regurgitation. Such granulomata have been reported in both the aortic and mitral valves and, in some cases, have required surgical intervention due to significant hemodynamic compromise [25]. Most previously reported cases of AR in RA involve moderate to severe regurgitation, typically accompanied by left ventricular dysfunction, congestive heart failure, and a clinical indication for aortic valve replacement. Case series and individual reports indicate a RA duration ranging from 8 to 19 years, with a predominance in female patients. These cases often required urgent hospitalization for heart failure or surgical management. The average age of affected individuals ranged from the late 40s to early 60s [26,27,28,29]. There are several well identified risk factors for VHD in RA, including prolonged RA duration, high inflammatory activity, male sex, the presence of subcutaneous nodules, and elevated inflammatory markers. Patients with disease duration exceeding 15 years have shown a higher prevalence of valvular heart lesions compared to those with shorter disease courses. The presence of subcutaneous nodules has also been associated with more severe valvular heart pathology, with nodulosis documented in up to 71.5% of RA patients presenting with valvular heart involvement [30]. Histopathological examination of valves affected by RA reveals a spectrum of changes, including non-specific inflammatory infiltration, granulomata, rheumatoid nodules, and calcification. These findings support the hypothesis that AR in RA may arise from both inflammatory and degenerative mechanisms [2]. In general, acute AR may results from various etiologies, including infective endocarditis, type A aortic dissection, trauma, annuloaortic ectasia, rheumatic fever, syphilitic aortitis, congenital bicuspid aortic valve, iatrogenic injury (e.g., following balloon valvuloplasty), and, rarely, spontaneous avulsion of the aortic valve commissures. In RA specifically, AR is thought to develop due to granulomatous thickening of the aortic valve cusps or diffuse fibrosis of the valvular heart apparatus [31]. Valvular heart lesions in RA typically lead to regurgitation, whereas those associated with rheumatic fever are more commonly stenotic [32]. Although VHD in RA is clinically silent in most cases, it can occasionally progress rapidly, leading to significant valve dysfunction. While some valvular granulomata may regress with corticosteroid therapy, accompanied by improvement in hemodynamic parameters, there are currently no established guidelines for the management of these cardiac manifestations in RA. This carries a potential risk of thrombus formation or superimposed infection, leading to infective endocarditis in the presence of nodular lesions on the cardiac valves [33]. The relationship between VHD and RA is not yet clearly established. In our case, AR may be explained by the presence of a bicuspid aortic valve, a congenital anomaly frequently associated with valvular dysfunction. Nevertheless, chronic systemic inflammation in RA, characterized by granulomatous lesions, fibrotic thickening, and valvular calcification, has also been proposed as a potential pathophysiological mechanism leading to valvular regurgitation [32,33]. Importantly, the present case occurred in the context of RA/SLE overlap syndrome, a particularly rare condition, which may further increase the risk of immune-mediated cardiac involvement. Therefore, the coexistence of bicuspid aortic valve and systemic autoimmunity highlights the complexity of attributing valvular disease in such patients to a single etiology. To provide a broader perspective, we reviewed the available literature on cardiac involvement in RA. Table 1 summarizes the main published articles, including study type, number of patients, cardiac manifestations, and key findings.

As shown in Table 1, valvular lesions have been documented in case series and case reports, often in patients with long-standing RA, while observational and review studies highlight vascular dysfunction and accelerated atherosclerosis as additional CV complications. Together, these findings indicate that RA-related cardiac involvement is heterogeneous and multifactorial, ranging from inflammatory valvular lesions to systemic vascular damage, and may act synergistically with congenital or degenerative mechanisms, as suggested in our case.

**Figure 4:**
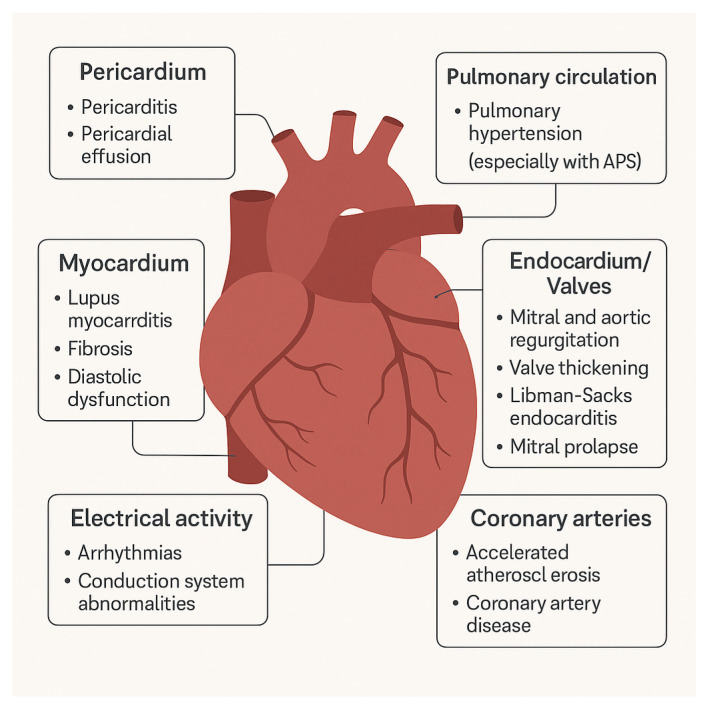
Cardiac disorders in systemic lupus erythematosus (SLE), including pericardial, myocardial, valvular, coronary, pulmonary, and conduction system involvement.

Valvular heart involvement is frequently observed in patients with SLE and represent clinically significant cardiac manifestation. Meta-analyses including over 500 patients with SLE have demonstrated a markedly increased prevalence of valvular abnormalities compared to healthy controls. The most commonly reported findings include mitral and aortic valve thickening, mitral regurgitation, and sterile vegetations, particularly affecting the mitral valve. Additionally, aortic and tricuspid regurgitation and mitral stenosis occur more frequently in SLE, whereas tricuspid thickening and aortic stenosis are less common [34]. Several echocardiographic studies have also documented valvular lesions in SLE patients independently of antiphospholipid syndrome. The most prevalent abnormalities include mitral valve thickening or vegetations, mitral valve prolapse, and aortic valve involvement, alongside regurgitation of the mitral, aortic, and tricuspid valves [35,36]. Furthermore, grade I left ventricular diastolic dysfunction and leaflet thickening associated with valvular regurgitation or intracardiac masses have also been reported [37,38]. Although some studies concluded that aPL do not significantly influence valvular lesion prevalence, other findings suggest their pathogenic role. For instance, a study from the 1992. emphasized the value of routine echocardiographic screening for early detection of frequently subclinical cardiac involvement and highlighted the possible contribution of aPL to valvular heart pathology [39]. More recent studies have demonstrated a strong association between aPL, especially anticardiolipin antibodies, and the development and progression of VHD in SLE. These antibodies have been implicated in mitral and aortic valve lesions and are associated with more severe outcomes, including significant regurgitation [40,41]. Importantly, SLE is recognized as an independent risk factor for VHD regardless of conventional CV risk factors. Additional predictors of VHD in SLE include prolonged disease duration, history of pericarditis, higher Systemic Lupus International Collaborating Clinics/American College of Rheumatology Damage Index scores, and the presence of Libman–Sacks endocarditis. These findings underscore the importance of routine echocardiographic surveillance, particularly in high-risk patients, to facilitate early diagnosis and prevent serious cardiovascular complications [42,43]. Figure 4 provides a schematic overview of the main cardiac disorders in SLE, including pericardial, myocardial, valvular, coronary, pulmonary, and conduction system involvement.

**Table 1:** Summary of published articles on rheumatoid arthritis (RA) and cardiac involvement.

References	Study Type	No. of Patients	Cardiac Involvement Described	Key Findings
[29]	Case series	12	Valvular lesions (mitral, aortic)	RA patients had granulomatous and fibrotic valvular lesions; associated with long disease duration.
[27]	Case series (5 cases)	5	Aortic regurgitation	All required surgical valve replacement; mostly female patients with long-standing RA.
[25]	Case report	1	Aortic regurgitation	Moderate-to-severe AR associated with RA; highlights role of inflammation.
[26]	Case report	1	Acute aortic regurgitation	Rapid progression within 2 weeks; required urgent surgical management.
[28]	Case report	1	Acute AR due to necrotizing granulomatous inflammation	Rare and life-threatening complication of RA.
[30]	Case report	1	Aortic regurgitation	Demonstrated fibrotic changes in aortic valve associated with RA.
[31]	Case report	1	Aortic regurgitation	RA-related AR diagnosed with advanced imaging; need for multidisciplinary management.
[43]	Systematic review	>40 studies	Accelerated atherosclerosis	Chronic inflammation in RA accelerates atherosclerosis and CV events.
[44]	Narrative review	-	Endothelial dysfunction, neurological extra-articular features	Links systemic inflammation with endothelial and vascular damage in RA.
[45]	Observational study	112	Vascular dysfunction	IL-17 identified as predictor of vascular impairment in RA.

AR, aortic regurgitation; CV, cardiovascular; IL, interleukin; RA, rheumatoid arthritis.

In addition to ECHO, advanced imaging techniques play an important role in assessing cardiac involvement in RA. Cardiac magnetic resonance (CMR) enables sensitive detection of myocardial inflammation, edema, and fibrosis, while providing precise measurements of ventricular function and volumes. Importantly, CMR can identify subclinical myocardial changes even in patients without overt cardiac symptoms, supporting its value in early risk assessment and monitoring of therapy response. Coronary computed tomography (CCT) is also useful, offering high-resolution, non-invasive evaluation of coronary plaque burden and morphology, which is particularly relevant given the increased risk of premature coronary artery disease in RA. Both the European Society of Cardiology and EULAR recognize CMR and CCT as valuable adjuncts in selected RA patients with unexplained cardiac symptoms or high CV risk, complementing echocardiography in comprehensive cardiovascular evaluation [15,16].

There is a multifactorial etiology underlying the increased CV disease burden in patients with RA, including VHD. In addition to traditional CV risk factors, chronic inflammation markers, such as ESR and CRP, the number of affected joints, disease activity and RA-specific autoantibodies collectively contribute to a central pathophysiological mechanism: accelerated atherosclerosis. Furthermore, pro-inflammatory cytokines including tumor necrosis factor-alpha (TNF-α), interleukin (IL)-17, IL-6, and IL-1β drive the development of atherosclerosis and vascular lesions by promoting a prothrombotic milieu characterized by endothelial dysfunction, insulin resistance, dyslipidemia, and altered activation of the coagulation cascade. These changes predispose to atherosclerotic plaque rupture and the onset of CV events. Chronic inflammation also disrupts the balance between nitric oxide release and other vasoactive substances, further contributing to endothelial dysfunction [44,45,46].

The underlying pathophysiological mechanisms contributing to the increased CV morbidity and mortality in SLE are broadly similar to those observed in RA. However, notable differences exist. In SLE, aPL antibodies play a pivotal role in promoting endothelial dysfunction, altering lipoprotein metabolism, and facilitating the development of unstable atherosclerotic plaques. In addition, both innate and adaptive immune dysregulation significantly contribute to vascular injury. Among cytokines, interferon-alpha has emerged as a central mediator in the immunopathogenesis of SLE and is strongly implicated in the acceleration of atherogenesis in this complex autoimmune condition. Additionally, prolonged exposure to glucocorticoids is recognized as a significant risk factor for CV system damage [45,46,47,48].

The pathogenesis of AR in autoimmune rheumatic diseases reflects complex interactions between immune-mediated injury, chronic inflammation, and structural remodeling of the aortic valve. In RA, pro-inflammatory cytokines, including TNF-α, IL-1β, IL-6, and IL-17, activate valve interstitial cells and macrophages, resulting in upregulation of matrix metalloproteinases (MMPs) and degradation of extracellular matrix proteins. These processes, together with the formation of pannus and granulomatous nodules within the valves, lead to valve thickening, fibrosis, and calcification [25,26,27,28,29,30,31,32,33,44,45,46]. Rheumatoid aortitis and accelerated atherosclerosis may further contribute to aortic root dilatation and valve insufficiency [30,31,44]. Valve involvement in SLE is primarily driven by immune complex deposition and complement activation on the endocardial surface, leading to Libman–Sacks vegetations and leaflet thickening. aPL further advances this process by inducing endothelial activation, stimulating platelet aggregation and thrombus formation, and accelerating fibrotic remodeling [34,35,36,37,38,39,40,41,42,43]. Recent studies highlight the role of type I interferon in destabilizing the valvular endothelium and perpetuating immune activation, thereby contributing to valvular scarring and regurgitation [47,48]. Despite these differences, both RA and SLE converge on common pathways: chronic cytokine-mediated inflammation, endothelial dysfunction, and matrix degradation. Thus, the immune response is central in both conditions, although granulomatous valvulitis predominates in RA, whereas endocardial damage mediated by immune complexes and aPL is typical of SLE. Understanding these molecular mechanisms not only explains clinical variability but also highlights the importance of tailored immunomodulatory therapy to reduce long-term CV complications.

In our case, the patient with rhupus had a degeneratively altered bicuspid aortic valve associated with moderate AR, while the left ventricle was of normal size with preserved systolic function. As there is currently no indication for surgical intervention, close clinical monitoring with cardiac ultrasound every six months is recommended, beginning with TTE and followed by TEE in the event of any deterioration in valvular function. In this patient, multiple interrelated factors likely contributed to the development of valvular pathology, although the precise pathophysiological mechanisms remain insufficiently elucidated. The coexistence of two distinct autoimmune diseases substantially increases the cumulative CV risk, reflecting the synergistic burden of systemic autoimmunity. Traditional CV risk factors, including arterial hypertension, hyperlipidemia, female sex, and active tobacco use, are further exacerbated by persistently elevated CRP and ESR, which may indicate suboptimal control of underlying chronic inflammatory activity. Although formal classification criteria for APS were not fulfilled, the presence of circulating aPL must be acknowledged as a potential contributor to endothelial dysfunction and thrombogenic risk. The chronicity of both autoimmune conditions further amplifies cumulative vascular injury. Additionally, intolerance to csDMARDs and discontinuation of RTX resulted in long-term glucocorticoid therapy as the mainstay of treatment. This reliance on steroids, combined with persistent systemic inflammation and traditional CV risk factors, likely amplified the overall burden on the CV system and may have contributed to the development of valvular pathology.

## 4. Conclusions

CVDs significantly influence the prognosis of patients with overlap syndromes, particularly those with concurrent RA and SLE, both of which independently confer increased CV risk. It should always be considered in the differential diagnosis of unexplained cardiac symptoms, especially in patients with long-standing RA/SLE. Importantly, cardiac involvement may also be present in asymptomatic individuals, underscoring the need for proactive screening. Regular follow-up, including periodic echocardiographic assessment, is essential to avoid missing subclinical valvular heart lesions. Aortic valve replacement should be considered when significant valvular dysfunction leads to severe regurgitation, as prognosis remains poor without surgical intervention. This case highlights the potential contribution of chronic systemic inflammation in RA/SLE to the development of AR, an extra-articular manifestation associated with unfavorable outcomes. These findings support the routine use of echocardiography in rheumatologic patients as a key strategy for early detection and prevention of life-threatening CV complications.

## Data Availability

Data are available on request to corresponding author.

## Abbreviations

The following abbreviations are used in this manuscript:

ACPA	Anti-cyclic citrullinated peptide antibodies
ANA	Antinuclear antibodies
anti-dsDNA	Anti-double-stranded deoxyribonucleic acid antibodies
anti-Sm	Anti-Smith antibodies
aPL	Antiphospholipid antibodies
AR	Aortic regurgitation
ARDs	Autoimmune rheumatic diseases
CCT	Coronary computed tomography
CMR	Cardiac magnetic resonance
CRP	C-reactive protein
csDMARDs	Conventional synthetic disease-modifying antirheumatic drugs
CT	Computed tomography
CVDs	Cardiovascular diseases
ECG	Electrocardiogram
ECHO	Echocardiography
ESC	European Society of Cardiology
ESR	Erythrocyte sedimentation rate
EULAR	European League Against Rheumatism
IL	Interleukin
LA	Lupus anticoagulant
MCTD	Mixed connective tissue disease
MSCT	Multi-slice computed tomography
MTX	Methotrexate
NT-proBNP	N-terminal pro b-type natriuretic peptide
PM/DM	Polymyositis/Dermatomyositis
RA	Rheumatoid arthritis
RF	Rheumatoid factor
RTX	Rituximab
SjS	Sjögren’s syndrome
SLE	Systemic lupus erythematosus
SSc	Systemic sclerosis
TEE	Transesophageal echocardiography
TNF	Tumor necrosis factor
TTE	Transthoracic echocardiography
VHD	Valvular heart disease
